# A Bayesian model for predicting monthly fire frequency in Kenya

**DOI:** 10.1371/journal.pone.0291800

**Published:** 2024-01-25

**Authors:** Levi Orero, Evans Otieno Omondi, Bernard Oguna Omolo

**Affiliations:** 1 Institute of Mathematical Sciences, Strathmore University, Nairobi, Kenya; 2 African Population and Health Research Center (APHRC), Nairobi, Kenya; 3 Division of Mathematics & Computer Science, University of South Carolina – Upstate, Spartanburg, SC, United States of America; 4 School of Public Health, Faculty of Health Sciences, University of the Witwatersrand, Johannesburg, South Africa; Jeonbuk National University, KOREA, REPUBLIC OF

## Abstract

This study presents a comprehensive analysis of historical fire and climatic data to estimate the monthly frequency of vegetation fires in Kenya. This work introduces a statistical model that captures the behavior of fire count data, incorporating temporal explanatory factors and emphasizing the predictive significance of maximum temperature and rainfall. By employing Bayesian approaches, the paper integrates literature information, simulation studies, and real-world data to enhance model performance and generate more precise prediction intervals that encompass actual fire counts. To forecast monthly fire occurrences aggregated from the Moderate Resolution Imaging Spectroradiometer (MODIS) data in Kenya (2000-2018), the study utilizes maximum temperature and rainfall values derived from global GeoTiff (.tif) files sourced from the WorldClim database. The evaluation of the widely used Negative Binomial (NB) model and the proposed Bayesian Negative Binomial (BNB) model reveals the superiority of the latter in accounting for seasonal patterns and long-term trends. The simulation results demonstrate that the BNB model outperforms the NB model in terms of Root Mean Square Error (RMSE), and Mean Absolute Scaled Error (MASE) on both training and testing datasets. Furthermore, when applied to real data, the Bayesian Negative Binomial model exhibits better performance on the test dataset, showcasing lower RMSE (163.22 vs. 166.67), lower MASE (1.12 vs. 1.15), and reduced bias (-2.52% vs. -2.62%) compared to the NB model. The Bayesian model also offers prediction intervals that closely align with actual predictions, indicating its flexibility in forecasting the frequency of monthly fires. These findings underscore the importance of leveraging past data to forecast the future behavior of the fire regime, thus providing valuable insights for fire control strategies in Kenya. By integrating climatic factors and employing Bayesian modeling techniques, the study contributes to the understanding and prediction of vegetation fires, ultimately supporting proactive measures in mitigating their impact.

## Introduction

Fires are a natural and integral part of many ecosystems in Kenya, particularly savannas and grasslands [1–3]. Historically, these ecosystems have relied on regular, low-intensity fires that help maintain ecosystem health and biodiversity [1, 4–7]. However, fire dynamics have shifted in in recent decades, with an alarming increase in both the frequency and intensity of these fires. These changes can be attributed to various factors including changes in land use practices, human activities, and climate change [2, 3, 8–11]. It is therefore crucial to use tools and techniques that allow prediction of fire occurrence thus allowing prevention of substantial losses [8, 12–14].

Understanding the problem of accurately predicting fire frequency based on historical fire and weather data is critical in this context [14]. By analyzing the relationship between past fire occurrences and the corresponding weather conditions, we can develop tools and techniques that enhance our ability to forecast and manage future fires. This study delves into the complexities of this issue, investigating the underlying factors that contribute to changes in fire patterns and emphasizing the importance of predictive methodologies. By shedding light on the challenges posed by evolving fire dynamics, we can pave the way for more efficient fire prevention and mitigation strategies in order to safeguard ecosystems, human lives, and valuable assets [14, 15].

The National Aeronautics and Space Administration (NASA) detects thermal activity on Earth via its satellite system called the Earth Observing System Data and Information System (EOSDIS). This system is part of the Earth Science Data program [16]. NASA’s Fire Information for Resource Management System (FIRMS) has gone through several iterations in order to improve its fire products, which are used by scientists in a variety of fields [17–19]. One of the products is the Moderate Resolution Imaging Spectroradiometer (MODIS). Researchers can analyze its data and cross-reference it with other climatic variables using specific and detailed country-level datasets [20–24]. MODIS fire data has been used in numerous studies on fire frequency and distribution all over the world [18, 19, 21, 23, 25, 26]. Rainfall and temperature are two important factors that influence the occurrence and frequency of fires [27, 28]. Data on these variables at the country level are available on several websites online, though at varying aggregation levels and formats [29, 30]. The interaction of rainfall and temperature has a significant impact on the fire regime and fire probability [31], which motivated the use of these variables in this study. By aggregating these data by month, it is possible to conduct research on the frequency of fires in a specific geographical region. Fire regimes vary due to weather changes and climate patterns, and the data show excessive dispersion, implying that it is critical to consider various techniques to deal with this feature. Tested models from simulation studies are applied to real-world fire frequency data in this study to evaluate performance on a larger scale.

In modeling fire occurrences, various techniques have been employed to predict fire frequency, particularly through discrete response regression models [32]. Among these models, count response models are widely regarded as the most suitable for datasets with discrete response variables in the form of counts. Commonly utilized count models include Poisson, Negative Binomial, Zero-Inflated, Zero-Truncated, Hurdle, and Random-effects models [33]. The foundational model in this category is the Poisson regression model, which serves as the basis for other related models. Count data often exhibit a right-skewed distribution, with the variance increasing proportionally to the mean of the underlying distribution [33]. To address situations where data display over-dispersion, an alternative type of count model is recommended [33]. Considering the well-established relationship between the mean and variance in Poisson and Binomial distributions, this study extends the Poisson generalized linear model (GLM) by incorporating an additional parameter to account for over-dispersion. By leveraging historical fire and weather data and employing Bayesian techniques, this work develops a robust predictive model for fire frequency, considering seasonal variations and addressing the potential over-dispersion in the data.

Several methods have been developed to deal with over-dispersion in count data in a variety of fields [34–37]. Unique features in the data, such as Markov patterns or the ability of a model to handle over-dispersion and under-dispersion have been explored [34, 37]. This study adds to the research that has been done on the statistical modeling of count response data with spatial and temporal features. Furthermore, it uses techniques that account for over-dispersion in count regression models [33, 38–40].

It is important to note that fires, whether caused by humans or natural causes, produce thermal anomalies that are detected by Earth’s satellites [17]. Specifically, humans clearing farmland or disposing of waste frequently cause massive fires that are unpredictable and difficult to predict. However, the relationship between temperature, rainfall, and fires is evident as higher temperatures and lower rainfall are typically associated with increased fire occurrence. As a result, during certain months of the year characterized by seasonal variations in rainfall and temperature, a substantial surge in the number of fires is observed [11, 27, 28, 41]. Several researchers have published their findings on the relationship between climate change, forest fires, and the impact on forests by the turn of the millennium [42]. Since then, several methods for predicting fire occurrences in various parts of the world have been developed, though they are limited to specific areas [21, 27, 41, 43–45].

While fires pose a significant threat to the environment, predictive tools are limited, and some studies have used historical fire data to build a probability-based model that indicated relative fire frequency rather than intensity [31]. Instead, count data on monthly fire frequency were used in this study. It expanded on the spatial term by incorporating climatic data specific to the fire’s location. Several authors have worked on different methods for predicting fires and their likelihood of occurrence. Several studies have found geo-statistical approaches to be useful in this prediction [21, 46, 47]. Lim et al. [21] compared the accuracy of satellite and field survey data on fire occurrence using a maximum entropy (MaxEnt) model in spatial modeling of fire probability. The satellite data used contained numerous detection errors thus reducing model accuracy. Although fire probability data is not used, this paper puts their recommendation to use spatial filtering to improve model accuracy into action.

Oliveira et al. [48] investigated fire occurrence in Mediterranean Europe and used random forest and multiple regression techniques to model its spatial patterns. The authors’ dependent variable in the model was fire density (number of fires per km^2^). The model data only covered the main fire season (June–September) thereby limiting the scope of the study and the impact of historical data on fire regimes. The present study uses modeling on data spanning 18 years to investigate and account for the potential effects of seasonality on prediction. Given that the number of fires in a given period is count data, some researchers decided to make predictions using Poisson processes [27, 49–52]. For the period 2007–2008, [49] used the number of forest fires by forest area as their response variable. This was a 24-month period, which introduced volatility because they admitted that their models underestimated the number of fires in months 2, 3, 8, and 9 of 2009, when there was an unusually high number of fires. This study acknowledges that predictions can only be accepted if the future behaves similarly to the past, and more data improves the model’s ability to make predictions.

Traditional regression techniques, on the other hand, have been used to predict fires with varying degrees of success [43]. Su et al. [53] used a geographically weighted Negative Binomial regression model, which outperformed a standard Negative Binomial regression model but did not fully account for seasonality. When there is over-dispersion (i.e., when the variance is greater than or equal to the mean), a negative binomial model is preferable [54]. Several methods are used to deal with count data features such as over-dispersion and auto-correlation. Avanzi et al. [32] modeled count processes using a Markov-modulated non-homogeneous Poisson process framework, extending the standard Markov-modulated Poisson process (MMNPP) model by using a more flexible approach that can capture both cyclical and non-recurring trends. By relaxing the intrinsic equi-dispersion assumption of Poisson regression, flexibility in modeling count data was introduced [33]. Given previous and current data on fires and weather data, a motivating problem for these Poisson-based models and the negative binomial model is forecasting the number of satellite-detected fires in a specific geographical region.

In this paper, a Bayesian Negative Binomial model for count data is developed and extended to include an informative time component. The selected count model is a good fit for the response variable, which is fire frequency. In addition, it allows incorporation of prior knowledge to improve predictions. Furthermore, it uses Bayesian inference, which provides a better intuitive framework to quantify uncertainty unlike other methods. Furthermore, the Bayesian Negative Binomial model is tested on real-world data to demonstrate its accuracy in predicting the number of future monthly and cumulative fires caused by these climatic variables. This allows for more accurate prediction intervals that can help in allocation of resources and planning of fire response strategies.

## Materials and methods

### Data sources

The data used in this study were extracted from different sources and were of two types; i) fire hot-spot data and ii) temperature and rainfall data.

#### Fire hotspot data

MODIS data on active fire pixels’ timing, location, and radiative power are stored in different formats [55]. We used the Collection 6 Level 1 Fire Products (abbreviated MCD14DL) in this case because these data sets provide image and geographic coordinates, fire pixel and mean background brightness temperatures, and fire radiative power (FRP) for each individual 1 km active fire pixel detected by MODIS [56]. The fires included were those presumed vegetation fire, active volcano, other static land source and offshore. Text files (.csv) from November 2000 to December 2018, containing fire hotspot data were downloaded and then combined to form a single file. The number of fires recorded for each month was then calculated, taking into account mean values of features such as fire radiative power, brightness, and time of day. However, only fires that were presumed to be vegetation fires caused by human activity were included in subsequent analyses.

#### Monthly climate data

**Fig 1:**
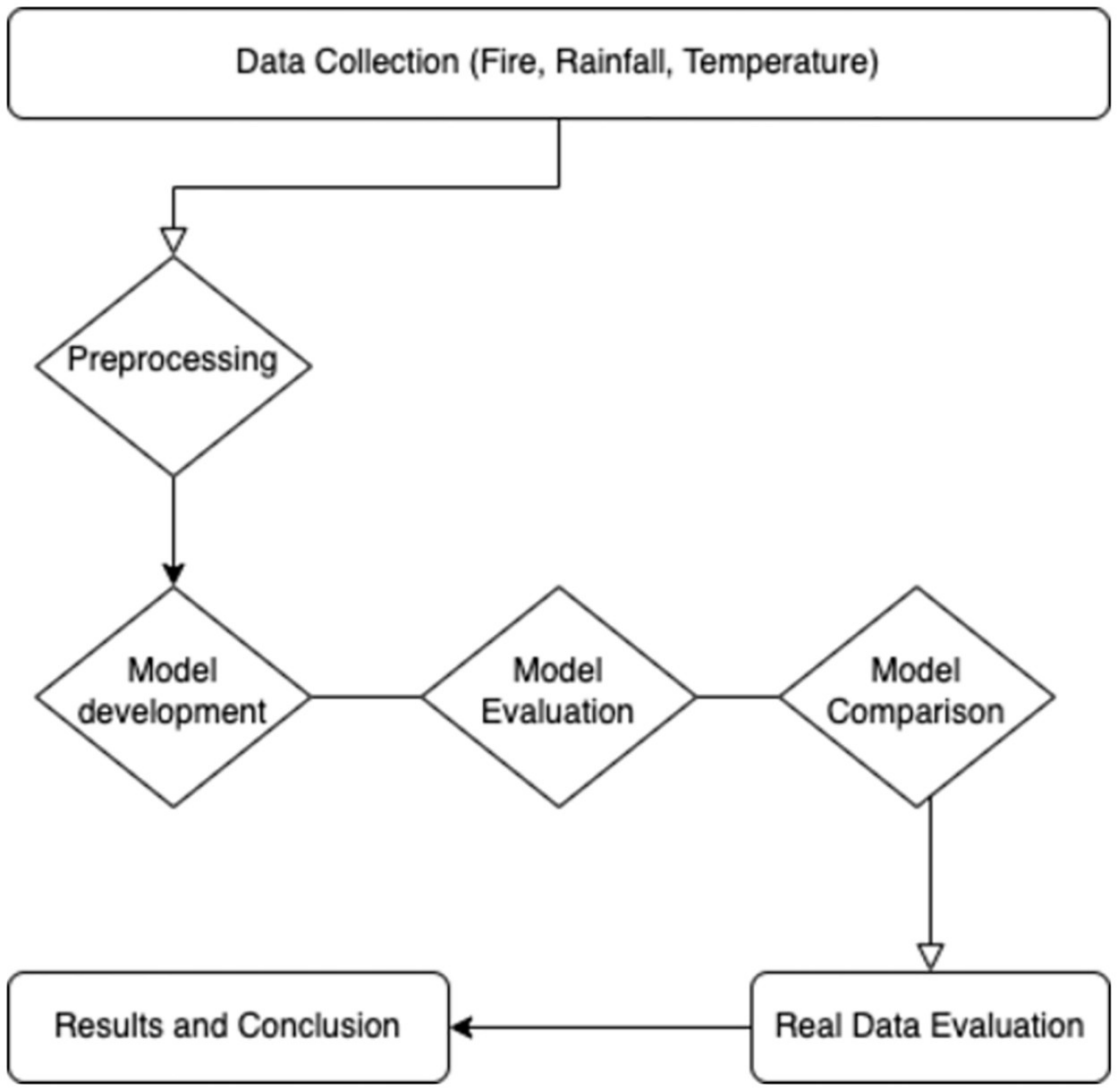
This methodological flowchart shows the approach used from data collection, preprocessing, modelling and presentation of results used.

This study made use of three monthly climatic variables: precipitation, minimum temperature, and maximum temperature. The precipitation (rainfall) and temperature data were obtained from WorldClim, a database of high spatial resolution global weather and climate data developed by the Climatic Research Unit at the University of East Anglia [57, 58]. The database contains historical data aggregated over a target temporal range of 1970–2018, based on data from 9,000 to 60,000 weather stations [57]. For each month of the year from the year 2000 to 2018, global GeoTiff (.tif) files were downloaded. The spatial resolution of the rainfall and temperature data available on the database is defined at three levels: approximately 21 km^2^ at the equator; around 85 km^2^; and approximately 340 km^2^ [57]. This study uses the level of 2.5 minutes, which corresponds to an area of approximately 21 km^2^ at the equator. These were then imported into QGIS 3.28.1 software [59], which was used to crop them to country using a Kenya .shp shape file. To identify the rainfall or temperature values for a specific fire hotspot, GPS coordinates were used as intersection points as they were available for both sets of data files. This was computed in R software version 4.1.0 using the packages raster, stars, sf, sp and stringr [60–64]. The final datasets were then written into the local directory for use in subsequent modeling. Fig 1 shows the methodological flowchart used in data collection, preprocessing and modeling framework. The variables in the rainfall and temperature datasets used in this study included month of year, year, rainfall amount in millimetres, minimum temperature and maximum temperature as variables.

While there was no explicit spatial variable in the model, it was ensured beforehand that, each fire hotspot was intersected with the corresponding temperature and rainfall value in that area at that time. This made the variables spatially valid.

### Simulation study

This simulation study aimed to evaluate the performance of two statistical models using simulated data sets with defined and different sample sizes in the presence of varying degrees of over-dispersion. The simulation study consisted of a total of 16 scenarios. The variables were simulated on *n* = 60, 120, 240, 360, which represented 5 years, 10 years, 20 years, and 30 years worth of monthly data. This study simulated 1000 datasets for each combination of parameters using R software version 4.1.0 and set the seed at “76568”.

To achieve the simulation of a dataset that was similar in characteristics to the real data, several steps were taken. First, the maximum temperature variable was assumed to follow a truncated normal distribution, given that the upper and lower bounds are known from the real data. Second, assuming a linear relationship between rainfall and temperature, a model was fitted on the two variables, and rainfall values were simulated to include random error terms. Lastly, the two simulated variables were fitted on to a standard negative binomial model with a specified initial *θ* to simulate a third response variable of the fire frequency, which is count data.

The mathematical formulation of a random number variable assumed to follow a truncated normal distribution. To generate random numbers from a truncated normal distribution, the lower and upper bounds to the range of possible values for *x* of the distribution, denoted by *a* and *b*, respectively are defined.

In this case, the lower bound *a* was 23.43 and the upper bound *b* was 34.82. The mean of the variable *μ* was 29.18 and its standard deviation *σ* was 2.29. This was implemented in the TruncatedNormal package in R.

To simulate the rainfall variable, initial mapping of the data showed a linear relationship between the two variables. To simulate rainfall data with a similar relationship with temperature, a simple linear regression model was fitted to the real data as as described in Eq (1).

$$\begin{array}{c} \text{Rainfall}=\beta_{0}+\beta_{1}\text{Maximum}\;\text{temperature}+\varepsilon , \end{array}$$ (1)

with the resulting model given by

$$\begin{array}{c} \text{Rainfall}=312.692-7.83\times \text{Maximum}\;\text{temperature}+\varepsilon \end{array}$$

To simulate rainfall values using the maximum temperature values, residuals of the model were calculated, then residual bounds were created with minimum and maximum residuals defined. Next, errors were simulated and assumed to follow a uniform distribution. Then, simulated values of rainfall were created by adding the simulated errors to the values of rainfall predicted by the regression model. Lastly, to simulate the fire counts, the two simulated variables were used as new data in predictions by a standard negative binomial fitted on the real data. The resulting model is given in (2).

$$\begin{array}{c} y∼\text{NegBinomial}(\mu =\phi ,\alpha =\;\text{exp}\left(\beta_{1}x_{1}+\beta_{2}x_{2}+\beta_{3}x_{3}+\beta_{4}x_{4}\right)) \end{array}$$ (2)

where *y* is the response variable and *μ* and *α* are the mean and dispersion parameters of the negative binomial model. Similar to the previous simulation, the residuals of the Negative Binomial model were calculated, residual bounds defined, errors simulated and then added to the values predicted by the model. These steps were implemented in a user-defined function in R that took four arguments; n = number of values to be simulated, theta = dispersion parameter in the NB model, mod = linear regression model object and data = real dataset. The output was a data frame. The synthetic covariate and response data were generated using a user-defined function, while varying sample size *n* and dispersion parameter *θ*. The subsequent analysis sought the best model for predicting the discrete count variable in the presence of an informative time component and over-dispersion.

This study evaluated two forms of the negative binomial models: a standard Negative Binomial model and a Bayesian Negative Binomial model. The simulation studies were used to evaluate the performance of the two statistical models [65]. Data were simulated, exported into Excel .csv files and then the two models were fitted and their performance evaluated. Because the data were time series, each dataset was split into a training and a testing dataset in a non-random and sequential 80:20 ratio. This means that in a dataset, the first 80 percent of the observations become the training set and the subsequent 20 percent are the testing set. Then, each model was fitted to the training dataset, and its predictive performance on both the training and test datasets was evaluated. Several metrics were used to assess model performance. Given the time series nature of the data, these included the Root Mean Square Error (RMSE), percent bias, and Mean Absolute Scaled Error (MASE). The model performance metrics were calculated using the Metrics package [66].

### Statistical models and estimation

#### Standard negative binomial model

The gamma mixture of the Poisson distributions is called the *negative binomial distribution* for *y*. Its probability mass function is

$$\begin{array}{c} p\left(y;\mu ,\phi \right)=\frac{\Gamma (y+\phi )}{\Gamma (\phi )\Gamma (y+1)}{\left(\frac{\mu }{\mu +\phi }\right)}^{y}{\left(\frac{\phi }{\mu +\phi }\right)}^{\phi },\;\;\;\;\;y=0,1,2,\ldots \end{array}$$ (3)

The fire hotspots counts *Y*_*i*_ were modelled using a negative binomial(NB) distribution in this case. The NB distribution has two parameters, the mean *μ* and a dispersion parameter *ϕ*:

$$\begin{array}{c} Y_{i}∼\text{NB}\left(\mu_{i},\phi \right) \end{array}$$ (4)

Using a logarithmic link function, the mean *μ*_*i*_ is specified as:

$$\begin{array}{c} \text{log}\left(\mu_{i}\right)=\beta_{0}+\beta_{1}\left({\text{Rainfall}}_{i}\right)+\beta_{2}\left(\text{Maximum}\;{\text{temperature}}_{i}\right)+\Theta_{i}+\varepsilon_{i} \end{array}$$

Θ_*i*_ is an collection of two pseudo covariates that make up an informative component:

$$\begin{array}{c} \Theta_{i}=\underset{\text{sine-cosine}\;\text{function}}{\underset{︸}{\beta_{3}\;\text{cos}\;(\frac{2p\pi }{{\text{time}}_{i}})+\beta_{4}\;\text{sin}\;(\frac{2p\pi }{{\text{time}}_{i}})}},\;\text{where}\;{\text{time}}_{i}=1,2,\cdots ,n\;\text{and}\;\text{p}\;=12 \end{array}$$

The raw real world data are often likely to be dominated by seasonal patterns and long term trends. To control for these patterns in the models, we add Θ_*i*_, which is some function of time, into the models [67, 68]. The error terms are assumed to be normally distributed:

$$\begin{array}{c} \varepsilon =\left(\varepsilon_{1},\cdots ,\varepsilon_{N}\right)∼N\left(0,\Sigma \right) \end{array}$$

The additional covariates Θ_*i*_ were included in the NB implementation on simulated data and were added during model fitting. To complete the description of the NB model, we specify the distribution of the dispersion parameter *ϕ*:

$$\begin{array}{c} \phi ∼Gamma(r_{j},d_{j}) \end{array}$$

which in general means that when X∼Gamma(r,d), the probability density *f*(*x*) is proportional to *x*^*r*−1^ exp(−*dx*). This model was implemented in R software using the glm.nb function in the MASS package [69]. The function performed a maximum likelihood estimation of the generalized Negative Binomial linear model.

#### Bayesian negative binomial model

In this type of model, at each loop stage of the estimation process, the respective prior distribution updates the posterior distribution for each predictor [70]. The posterior distribution in (3) is updated by multiplying the likelihood by the prior:

$$\begin{array}{c} p(\theta |y)\propto L(\theta )\pi (\theta ) \end{array}$$ (5)

*p*(*θ*|*y*) is the posterior distribution that explains the predictors; *L*(*θ*) is the likelihood function and *π*(*θ*) is the prior distribution [70]. Assuming the Negative Binomial characteristics of the previous model in (3), the Bayesian Negative Binomial model takes the form described in Eq (6).

$$\begin{array}{c} y_{i}∼\text{NB}\left(\mu_{i},\nu_{i}\right). \end{array}$$ (6)

The inverse link function can be expressed as

$$\begin{array}{c} \mu_{i}=\;\text{exp}\left(x_{i}^{\prime }\alpha \right)\mu_{i}=\;\text{exp}\left(\alpha_{0}+\alpha_{1}x_{1}+\alpha_{2}x_{2}+\alpha_{3}x_{3}+\alpha_{4}x_{4}\right), \end{array}$$ (7)

where *α*_0_, *α*_1_, *α*_2_ *α*_3_, and *α*_3_ are the model parameters and *ϕ* is the dispersion parameter. Using a logarithmic link function, the mean *μ*_*i*_ becomes

$$\begin{array}{c} \text{log}\left(\mu_{i}\right)=\alpha_{0}+\alpha_{1}\left({\text{Rainfall}}_{i}\right)+\alpha_{2}\left(\text{Maximum}\;{\text{temperature}}_{i}\right)+\Theta_{i}+u_{i}, \end{array}$$ (8)

where Θ_*i*_ is an collection of two pseudo covariates that make up an informative time component, similar to the NB model in the previous section given by

$$\begin{array}{c} \Theta_{i}=\underset{\text{sine-cosine}\;\text{function}}{\underset{︸}{\beta_{3}\;\text{cos}\;(\frac{2p\pi }{{\text{time}}_{i}})+\beta_{4}\;\text{sin}\;(\frac{2p\pi }{{\text{time}}_{i}})}},\;\text{where}\;{\text{time}}_{i}=1,2,\cdots ,n\;\text{and}\;\text{p}\;=12 \end{array}$$ (9)

The informative time component made up of the time term and the sine-cosine function is important to account for the seasonality and cyclic characteristics of the data [67, 68]. Then, the distribution of the dispersion parameter *ν*_*i*_:

$$\begin{array}{c} \nu_{i}∼Gamma\left(f_{j},r_{j}\right) \end{array}$$ (10)

which in general means that when X∼Gamma(f,r), the probability density *f*(*x*) is proportional to *x*^*f*−1^ exp(−*rx*). The covariate coefficients *α*_0_, *α*_1_, *α*_2_, *α*_3_, *α*_4_ and *α*_5_ have the following prior distributions:

$$\begin{array}{c} \alpha_{m}∼N(0,\frac{1}{\tau \alpha_{m}}),\;\;m=0,1,2,3,4. \end{array}$$ (11)

This represents a weak prior belief about the values of the model parameters. The prior for the dispersion parameter *ν*_*i*_ is a Gamma distribution with small shape and rate parameters, representing a vague prior belief about the value of *ν*_*i*_. This model was implemented in R software using the stanglm.nb function in the rstanarm package [71]. The function performed a full Bayesian estimation of the generalized Negative Binomial linear model via Markov Chain Monte Carlo sampling algorithm. The outcome’s posterior predictive distribution was used as the source of a single draw of the predictions used to calculate model performance.

## Ethical approval

The data used did not have any personal identification data and the Strathmore University Ethics Review Committee approval was obtained.

## Results and discussion

### Simulation results

Simulations were carried out under different sample sizes (n) and varying degrees of the dispersion parameter (*θ*). The sample sizes used were 60, 120, 240 and 360 months while the *θ* values were 1.5, 5, 10 and 100. For each combination, the model performance of the Negative Binomial (NB) and the Bayesian Negative Binomial (BNB) are analysed and represented. The metrics used are bias on the testing datasets, Mean Absolute Scale Error (MASE) on the testing datasets, and Root Mean Squared Error (RMSE) on both the testing and training datasets. Table 1 shows the results obtained.

**Table 1:** Model performance metrics of the simulated number of datasets for each dispersion and sample size combination.

*θ*	*n*	BNB_bias_	NB_bias_	BNB_mase_	NB_mase_	BNB_rmse1_	NB_rmse1_	BNB_rmse2_	NB_rmse2_
1.5	60	-0.404	-4.555	0.037	0.051	4.083	29.142	4.373	5.988
1.5	120	0.899	-11.245	0.032	0.144	3.791	30.111	2.825	11.729
1.5	240	0.803	-10.492	0.029	0.171	3.889	35.434	2.070	10.969
1.5	360	0.713	-9.942	0.029	0.176	3.900	36.598	1.929	10.405
5.0	60	-0.405	-4.555	0.037	0.051	4.084	29.142	4.374	5.988
5.0	120	0.753	-11.498	0.030	0.147	3.751	30.260	2.675	11.978
5.0	240	0.817	-10.441	0.029	0.172	3.862	35.233	2.089	10.917
5.0	360	0.738	-9.901	0.029	0.175	3.863	36.464	1.930	10.369
10.0	60	-0.403	-4.554	0.037	0.051	4.083	29.142	4.376	5.988
10.0	120	0.899	-11.245	0.032	0.144	3.790	30.111	2.825	11.729
10.0	240	0.803	-10.492	0.029	0.171	3.888	35.435	2.071	10.969
10.0	360	0.714	-9.942	0.029	0.176	3.901	36.598	1.929	10.405
100.0	60	-0.405	-4.554	0.037	0.051	4.084	29.142	4.374	5.988
100.0	120	0.753	-11.498	0.030	0.147	3.752	30.260	2.674	11.979
100.0	240	0.004	-10.441	0.721	0.172	31.841	35.233	32.536	10.917
100.0	360	0.739	-9.819	0.029	0.174	3.840	36.161	1.940	10.287

According to results in Table 1, the negative binomial mostly outperformed the BNB model in terms of the model percent bias, while the Bayesian negative binomial was a better model in terms of the MASE on the testing dataset, RMSE on the training datasets (rmse1), and RMSE on the testing datasets(rmse2). For instance, at *θ* = 1.5 and *n* = 60, the bias was -4.56 for the negative binomial model, which was lower compared to the bias of -0.40 for the Bayesian negative binomial model. The trend was similar at *θ* = 5 and *n* = 360, where the negative binomial model had a lower bias of -9.90. Further, the same is the case when sample is of *θ* = 100 and sample size *n* is 240 where the bias of the Bayesian negative binomial model (bias = 0.004) is higher than that of the negative binomial model under the same conditions (bias = -10.441). Looking at the MASE,Table 1 shows how the values are lower for the Bayesian negative binomial model. For instance, at *θ* = 1.5 and *n* = 60, the MASE is 0.037 for the BNB model and 0.051 for the negative binomial model. When at *θ* = 10 and *n* = 360, the BNB model outperforms the NB model with a lower value of MASE = 0.029. For the RMSE on training data, when *θ* = 1.5 and *n* = 60, the value of the metric is lower for the BNB model (4.083) suggesting that it is a better model. This is similar across the board for the different sample sizes and values of *θ*. Finally, this is the same for the RMSE on testing data as seen in the 9th column of 1, where RMSE is 4.373 for the BNB model and 5.988 for the NB model when *θ* = 1.5 and *n* = 60.

**Fig 2:**
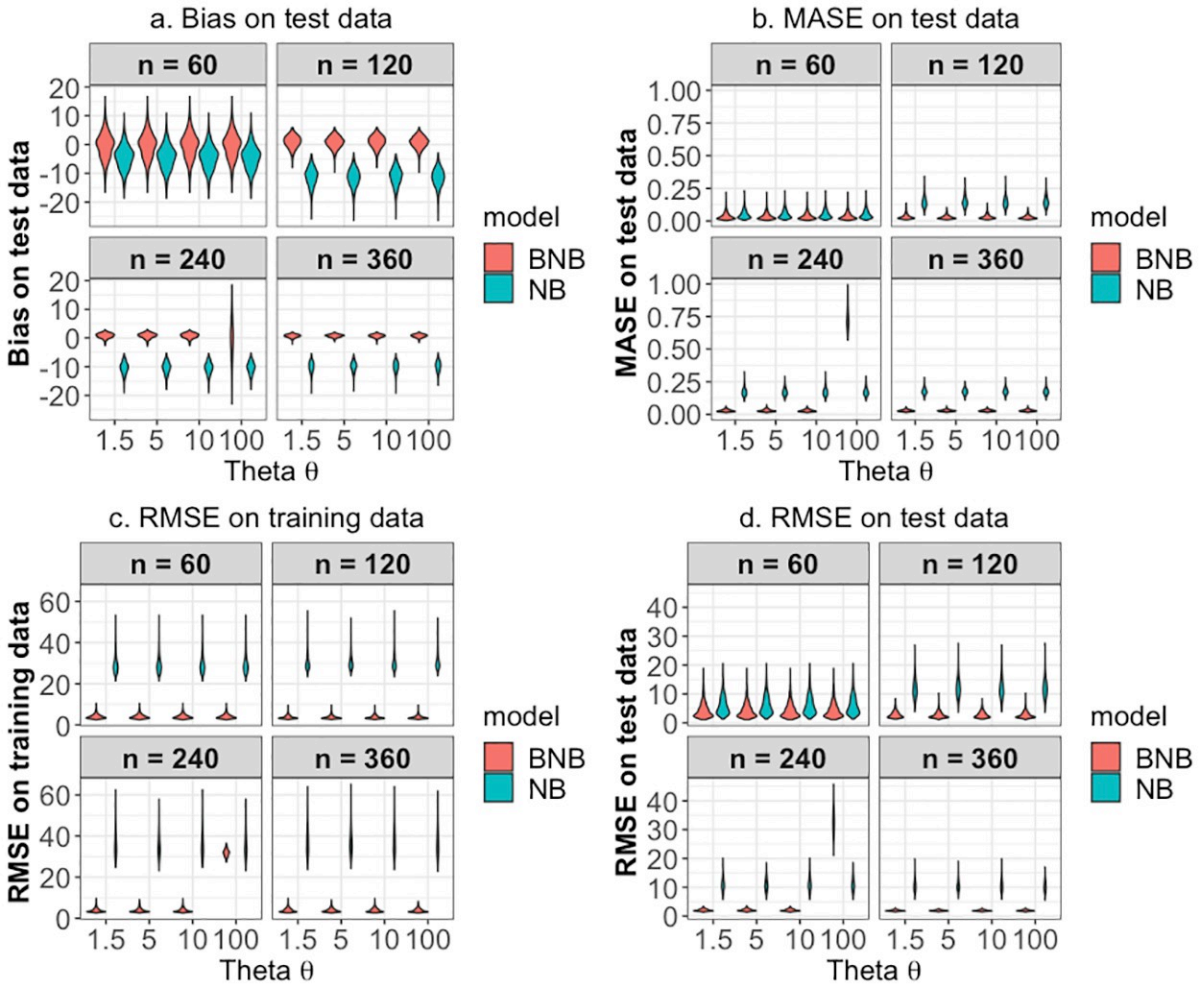
Violin plot of the four model performance metrics in the simulation study. Panel **a** shows the model results by bias on test data, Panel **b** shows the model results by MASE on test data, Panel **c** shows the model results by RMSE on training data and Panel **d** shows the model results by RMSE on test data.

Overall, the BNB model becomes better (lower error) when sample size (*n*) increases, suggesting that when more training data is available, the predictive performance improves. This is is also visible in the distribution as shown by the violin plots in Fig 2. For all the metrics, it is expected that a lower metric value is representative of better predictive performance. The length of the violin plot represents the range of values, while the width indicates the density of the data at different levels along the y-axis. For the plot a in Fig 2, the BNB violin plots appear higher than the negative binomial model plots, suggesting that there is a greater concentration or frequency of bias data points in that region and thus, worse performance. This is different for the other three plots b, c and d, in which the BNB model shows lower error values and thus better performance.

### Results on actual data

#### Descriptive statistics

Table 2 shows the summary statistics of the data and the selected variables where the sample size is 218. The overall minimum number of fires over the period (2000–2018) was 10 while the maximum number was 1661. In the period of 218 months, the mean and median are 277.13 and 155.00, respectively, with a standard deviation of 304.91. On the other hand, the mean and median mean maximum temperatures were 29.19 and 29.29 respectively. For the mean minimum temperature, the mean was 18.17 degrees Celsius with a standard deviation of 2.14. Lastly, the mean amount of rainfall was 84.04mm with a standard deviation (SD) of 51.69.

**Table 2:** Summary of real world dataset variables (n = 218).

Variable	Minimum	Maximum	Median	Mean	SD
Fire frequency	10.00	1661.00	155.00	277.13	304.91
Maximum temperature	23.44	34.82	29.27	29.19	2.30
Minimum temperature	11.26	22.34	18.36	18.17	2.14
Rainfall	8.43	297.86	70.66	84.04	51.69

**Fig 3:**
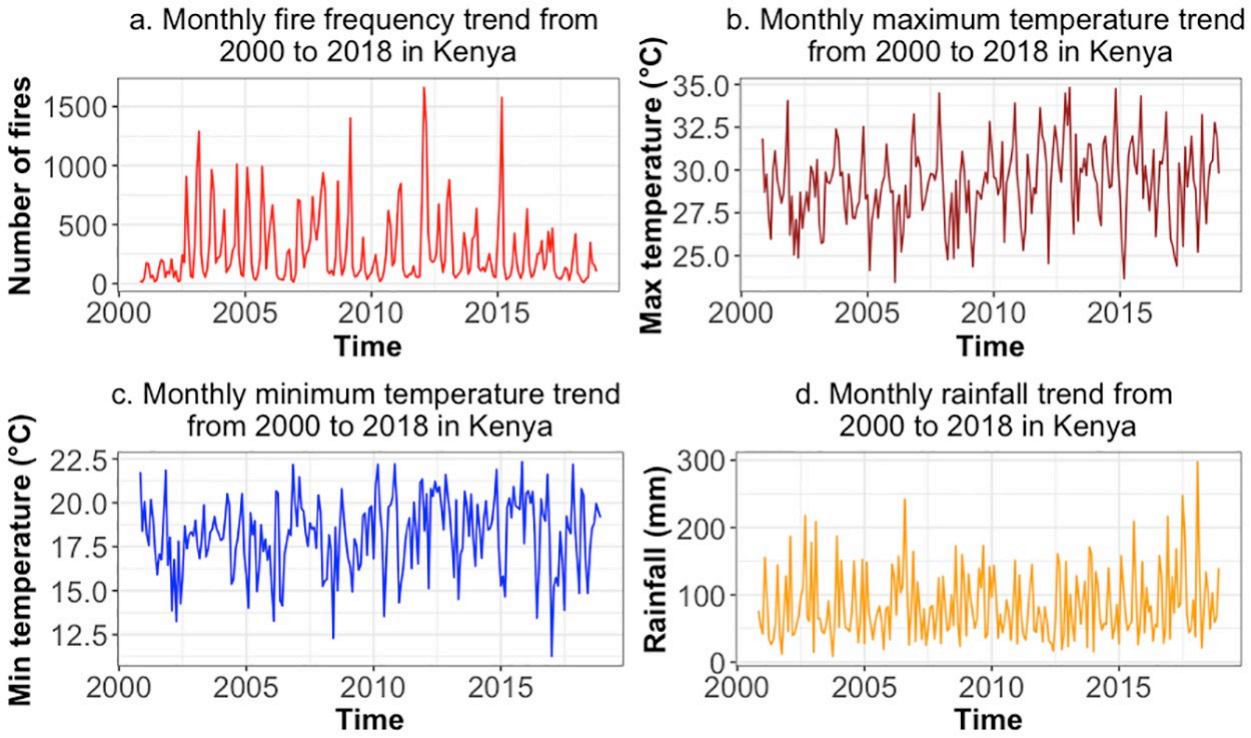
Time series of the four variables in the study from the real world data. Panel **a** shows the time series of monthly fire frequency, Panel **b** shows the monthly maximum temperature, Panel **c** shows the monthly minimum temperature and Panel **d** shows the monthly rainfall amounts.

Fig 3 shows the trend and patterns observed on the data and its four variables between the year 2000 and 2018. In Panel (a) of Fig 3, there are irregular patterns with the number of fires being high at certain times of the years and low in others. The highest number of fires was above 1500 in a month. In In Panel (b) of Fig 3, there appears to be a similar irregularity and there average monthly maximum temperatures have been rising over time. In Panel (c) of Fig 3, there is a similar trend as described in Panel (b) of Fig 3, which suggests that on average, minimum temperatures have been rising in the period. Lastly, the results suggest that there seem to be an increase in the average rainfall from 2015 as shown in Panel (d) of Fig 3. Furthermore, the results suggest that there are visible fluctuations that can be expected to happen within a calendar year. The irregular patterns in each graph show the presence of seasonality in the data, which is to be expected as fires, rainfall and temperature change with season of the year.

**Fig 4:**
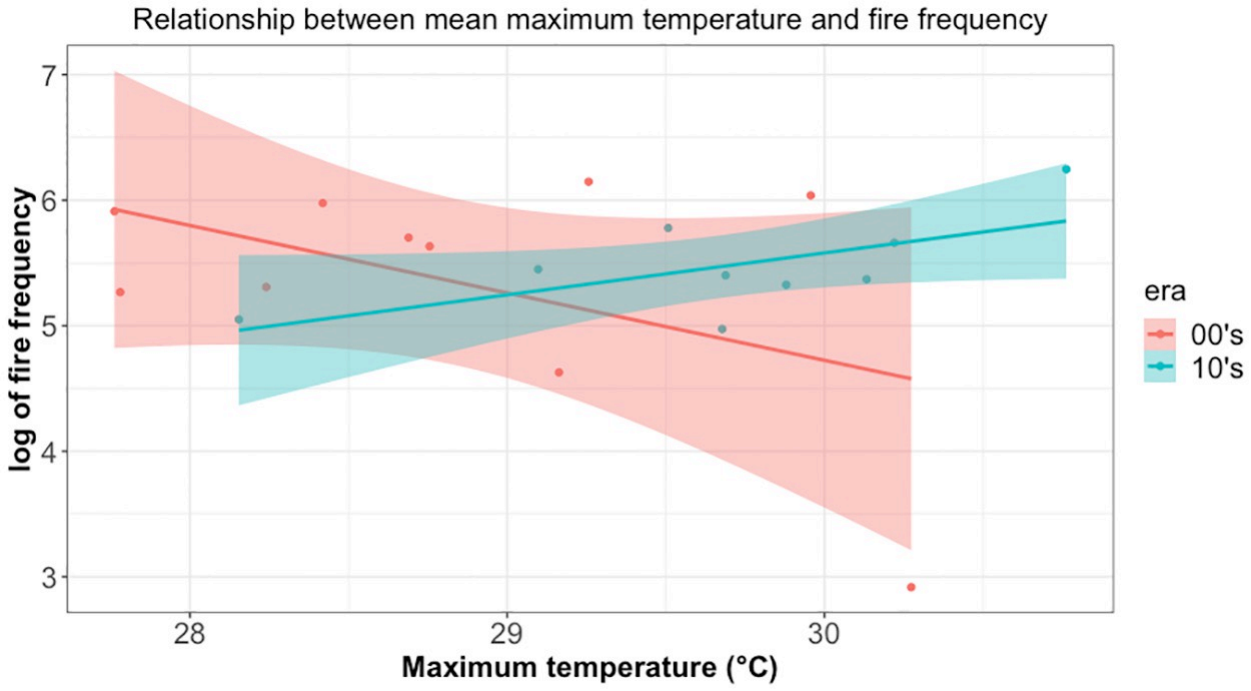
Scatterplot of relationships between mean annual maximum temperature and fire frequency. Graphs show different relationship for each era suggesting the difference in time periods and effect of other variables.

**Fig 5:**
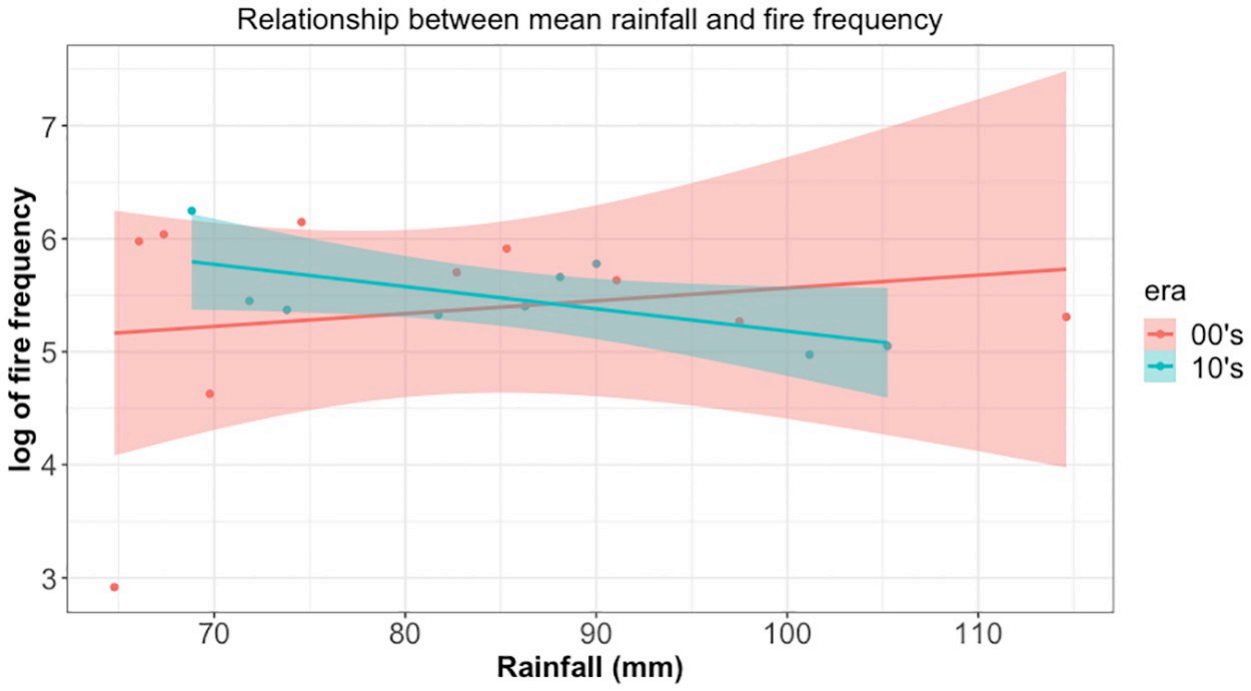
Scatterplot of relationships between mean annual rainfall and fire frequency. Graphs show different relationship for each era suggesting the difference in time periods and effect of other variables.

The relationship between maximum temperature and fire frequency was irregular (See S1 Fig). In March, the two variables show a strong inverse relationship and this suggests that an increase in maximum temperature is associated with fewer fire incidences. On the other hand, this is an opposite behaviour in January, February, May, June, October and December. In these months, an increase in maximum temperature is associated with more fire incidences. The other months also have specific trends though suggest weak relationships. The relationship between rainfall and fire frequency was also irregular by month (See S2 Fig). Here, there seems to be a pattern suggesting an inverse relationship between the two variables. This means that for all months except September and December, more rainfall is associated with fewer vegetation fires. To get more information from the data on the relationship between these variables, we introduced a dummy variable called era, which took two values: 00’s if data were from the 2000–2009 period and 10’s if the data were from the 2010–2018 period. The relationship between maximum temperature and fire frequency is shown in Fig 4. Here, there seems to be a pattern suggesting an inverse relationship between the two variables in the 00’s and a direct relationship between the two variables in the 10’s period. This suggests that since the 10’s higher maximum temperature is associated with more vegetation fires, contrary to the previous period. The relationship between rainfall and fire frequency is shown in Fig 5. Here, there seems to be a pattern suggesting a direct relationship between the two variables in the 00’s and an inverse relationship between the two variables in the 10’s period. This suggests that since the 10’s more rainfall is associated with fewer vegetation fires, contrary to the previous period.

#### Model performance on actual data

Both models were fitted on real world data and the results evaluated as shown in Table 3. The results show that the BNB outperformed the NB on the RMSE on training set, RMSE on the testing set, MASE on the testing set and the percentage bias on the testing set as shown in Table 3.

**Table 3:** Model performance metrics on data with n = 218.

Metric	Negative Binomial	Bayesian Negative Binomial
RMSE on training set	308.88	307.58
RMSE on testing set	166.67	163.22
MASE on testing set	1.15	1.12
Bias on testing set	-2.62%	-2.52%

For the training set, the Bayesian Negative Binomial model achieved a slightly better performance (RMSE = 307.58) as compared to the traditional Negative Binomial regression (RMSE = 308.88). This superiority of the Bayesian approach was also evident in the testing set, where the Bayesian Negative Binomial model resulted in an RMSE of 163.22, whereas the Negative Binomial model exhibited an RMSE of 166.67. On the testing set, both models exhibited closely comparable performance, with MASE values of 1.15 and 1.12 for the Negative Binomial and Bayesian Negative Binomial models, respectively. The bias in predictions on the testing set was found to be slightly less for the Bayesian Negative Binomial model (-2.52%) in comparison to the traditional Negative Binomial model (-2.62%).

Table 4 summarizes the beta estimates of the Bayesian Negative Binomial Model and associated 95% credible intervals for the predictors applied to the data. The estimated baseline frequency, when all other predictors are zero, is 7.69, with the associated 95% credible intervals extending from 4 to 10.19. In summary, the BNB model indicates significant effects of the max temperature and sine function on the response variable. The influences of mean rainfall and the cosine function, however, are statistically uncertain suggesting possibility of interaction terms and unexplained variance from other variables.

**Table 4:** BNB model beta estimates and credible intervals on data.

Term	Estimate	Standard error	CI-2.5%	CI-97.5%
(Intercept)	7.69	1.55	4.00	10.19
Max temperature	-0.09	0.05	-0.18	-0.00
Mean rainfall	0.002	0.002	-0.002	0.006
Sine function	0.83	0.19	0.43	1.19
Cosine function	-0.05	0.17	-0.40	0.27

**Fig 6:**
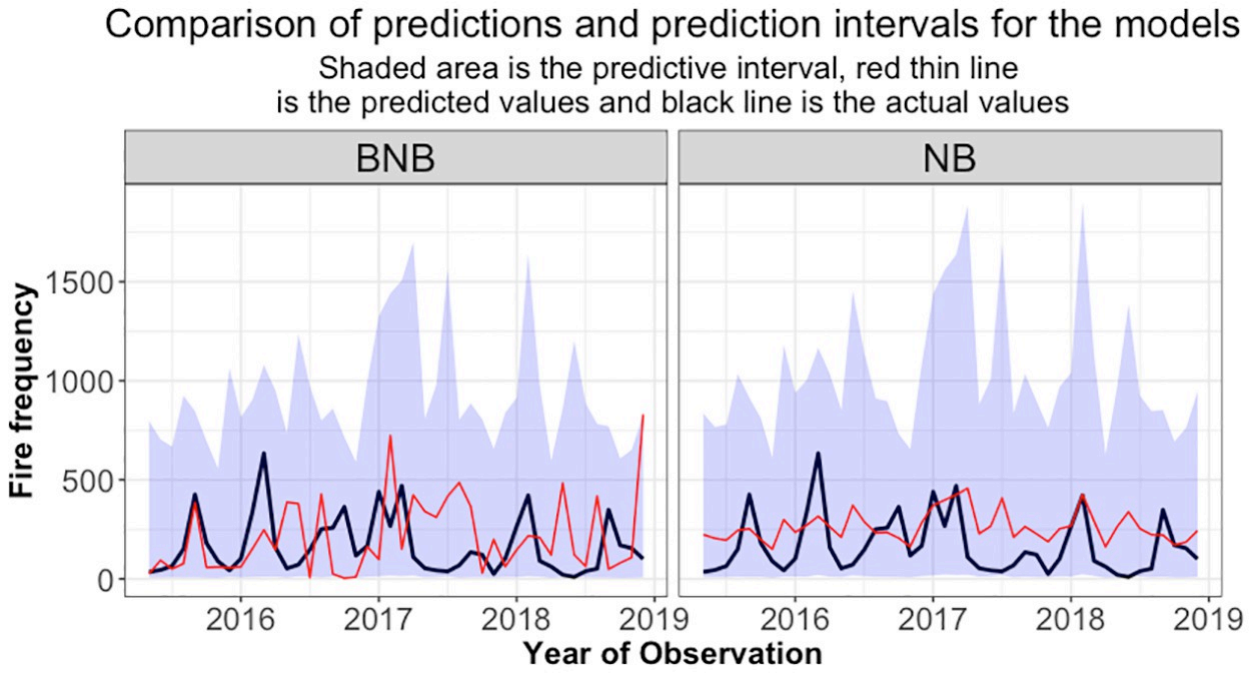
The actual fire counts, the predicted values and the prediction intervals of the NB and BNB models on testing data. Desired probability is 0.9.

To better understand the significance of prediction intervals, the NB and BNB models were used to calculate prediction intervals on the test dataset. Fig 6 depicts these outcomes. It can be seen that the actual values are mostly within the prediction interval with the deviation from the upper prediction intervals being slightly larger for the NB model. While both models seem to have a similar pattern and trend, the predicted values of the BNB seems to be a better fit as they have a larger variance. It can be said that if sampling process was repeated indefinitely, 95 percent of the predicted intervals would include the new observation. The correlation between the BNB’s observed fire counts in the test data and predicted counts was not statistically significant, suggesting a weak non-linear relationship, r = -0.07, t(42) = -0.45. On the other hand, the correlation between the NB model’s observed fire count in the test data and and the predicted values was statistically significant, indicating a moderate positive relationship, r = 0.32, t(42) = 2.19. This indicates that the relationship between the observed and predicted values is not well captured by a linear association.

The seasonal cycle is visible in the time series plots of the four variables of fire frequency, minimum temperature, maximum temperature, and rainfall. The simulation results show that both models perform well on overdispersed data, with the BNB model outperforming the Negative Binomial (NB) in terms of Mean Absolute Scaled Error (MASE), Root Mean Squared Error (RMSE) on testing sets, and RMSE on training sets. Furthermore, when applied to real data, the BNB model outperformed the NB model for all the four metrics. While the simulations were designed to handle some of the complexities found in real-world data, they clearly did not cover all potential complications.

However, it should be acknowledged that while the simulations addressed some complexities in real-world data, they did not encompass all potential complications. Real-world environmental variables may possess diverse autocorrelation structures, which the BNB model seems to handle better than the NB model. The empirical findings are limited to the specific case studied, and future research should explore and address data complexity while confirming the results through validation with real data, employing more appropriate simulation methods.

The results show how different periods in a time series can have different patterns, making any underlying relationships difficult to establish. For instance, the relationships between fire frequency and rainfall or temperature vary by month, potentially influenced by climatic factors, farming seasons, or human activities. Moreover, variations are observed when analyzing the data by decade or time period. Notably, there has been a shift in the relationship from 2010 to 2018, suggesting an increasing impact of climate change, particularly in higher temperatures, which are associated with more fires. The BNB model also provided prediction intervals that were close to the actual predicted values. This feature is valuable as large prediction intervals without nearby real data would lack practical significance. In the real data, MASE values greater than 1 for both models indicate that the actual forecasts performed worse than a superior time series model in terms of mean absolute error. Achieving MASE values below 1 may have been challenging due to structural characteristics of the data.

In the context of count data, a discrete probability distribution like Poisson or Negative Binomial is the most appropriate representation to avoid bias and misspecification in the model [72]. Given the time-related nature of the data, time-trend analysis plays a crucial role in understanding system dynamics and changes. The authors acknowledge the complexity of predicting the number of fires due to various influencing factors [73]. However, due to the randomness of the number of fire occurrences each month, application of the Bayesian model was more appropriate [74, 75]. The value offered by the Bayesian technique was the assumption that the parameter of interest is random, and that it lies within the prediction intervals [74]. In many instances, the BNB model generated results similar to that of the frequentist NB model [74, 75]. The intention was to allow for more precise predictions through this method as reported by [76], which the results in this study have achieved.

A problem this study sought to solve was on the time dimensions of data used to predict fire frequency. Unlike the shorter time spans used in [48–50], this study simulated data under different scenarios and thus had sample sizes of 60, 120, 240 or 360 months. As is utilised in literature, the application of the Negative Binomial distribution as in [43, 53, 54] allowed better modeling of over-dispersed count data. To tackle the issue of under-predictions as in [49], the implementation of the Bayesian Negative Binomial model allowed for the calculation of better prediction intervals. This is coming up as a useful technique as presented by [77, 78] who acknowledge the importance of Bayesian inference in fire prediction. This study contributes to understanding fire frequency prediction, highlighting the superiority of the Bayesian Negative Binomial model in handling overdispersed data and providing reliable prediction intervals. However, further research is necessary to explore and account for data complexity, validate the findings with real data, and refine simulation methods.

## Conclusion

This study utilized the assumption that the occurrence of fire incidents followed a Poisson process. Although the results are intriguing, they do not provide practical insight into predicting the timing of future events. Additionally, the absence of outliers in the simulation runs is inconsistent with real-world systems, where outliers would be expected. Nevertheless, the fitted models offer valuable guidance on the behavior of real-world variables. It is important to note that the fire regime system is complex, influenced by various environmental and human factors, and cannot be entirely explained by the variables used in this study. It is also noted that although historical data shows variation in fire behaviour over two time periods analysed (2000–2009 (00) and 2010–2018 (10)), these have not been included in the simulations.

To enhance the comprehensiveness of this study, several avenues for expansion can be pursued. First, additional variables that may impact the fire regime could be incorporated. Furthermore, the inclusion of outliers in the simulated datasets would better reflect real-world scenarios. Additionally, the proposed modeling framework can be extended to include predictions of monthly fire frequency for shorter time horizons (for example, less than 6 months ahead). Refining the proposed models to optimize performance metrics such as MASE and RMSE on the testing datasets would also be beneficial. Incorporating polynomials into the model specification might offer a way to improve the models.

In conclusion, this study highlights the potential for further statistical modeling of count response data with spatial and temporal features. The authors recommend the use of the Bayesian Negative Binomial model, which accounts for and addresses overdispersion, as the preferred model for real data. Although not exhaustive, this work provides valuable insights into the behavior of models under different conditions, leading to more comprehensive predictions. Ultimately, these predictions can contribute to effective fire planning and mitigation efforts in Kenya.

## Supporting information

S1 Fig The relationship between maximum temperature and fire frequency. Scatter-plots of monthly relationships between maximum temperature and fire frequency. Graphs show different relationship for each month suggesting the seasonality and effect of unknown variables.
(TIF)

S2 Fig The relationship between rainfall and fire frequency. Scatter-plots of monthly relationships between mean rainfall and fire frequency. Graphs show different relationship for each month suggesting the seasonality and effect of unknown variables.
(TIF)
