# Supplementary figures and images for: A Bayesian model for predicting monthly fire frequency in Kenya
Source: PLoS One. 2024 Jan 25;19(1):e0291800. doi: 10.1371/journal.pone.0291800 (PMC10810550; doi:10.1371/journal.pone.0291800)

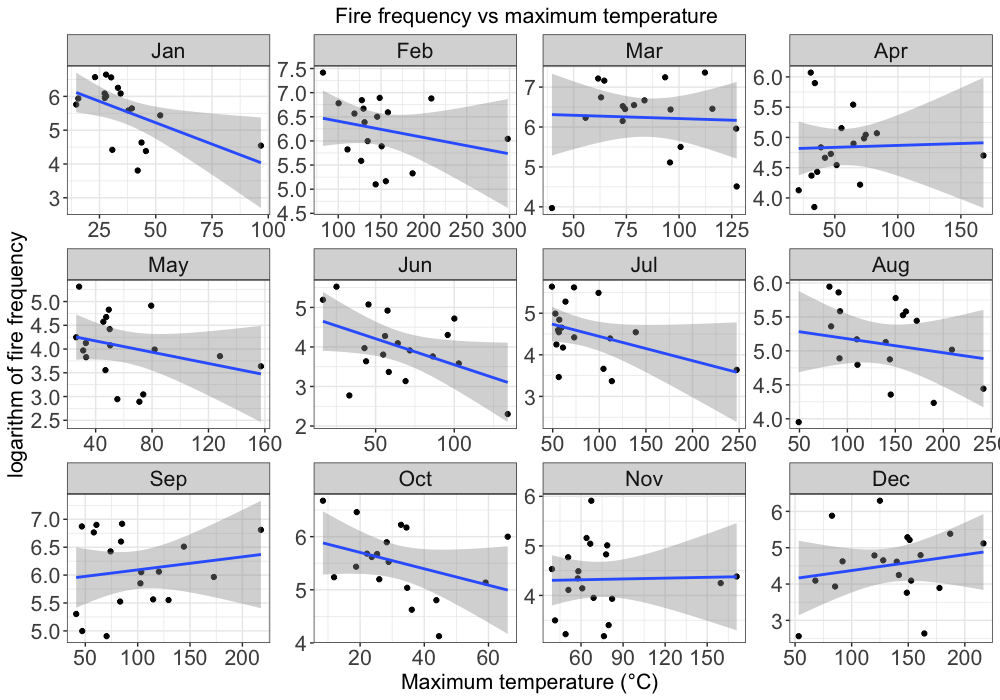

Supplement: S1 Fig — Scatter-plots of monthly relationships between maximum temperature and fire frequency. Graphs show different relationship for each month suggesting the seasonality and effect of unknown variables. (TIF) [file pone.0291800.s001.tif]

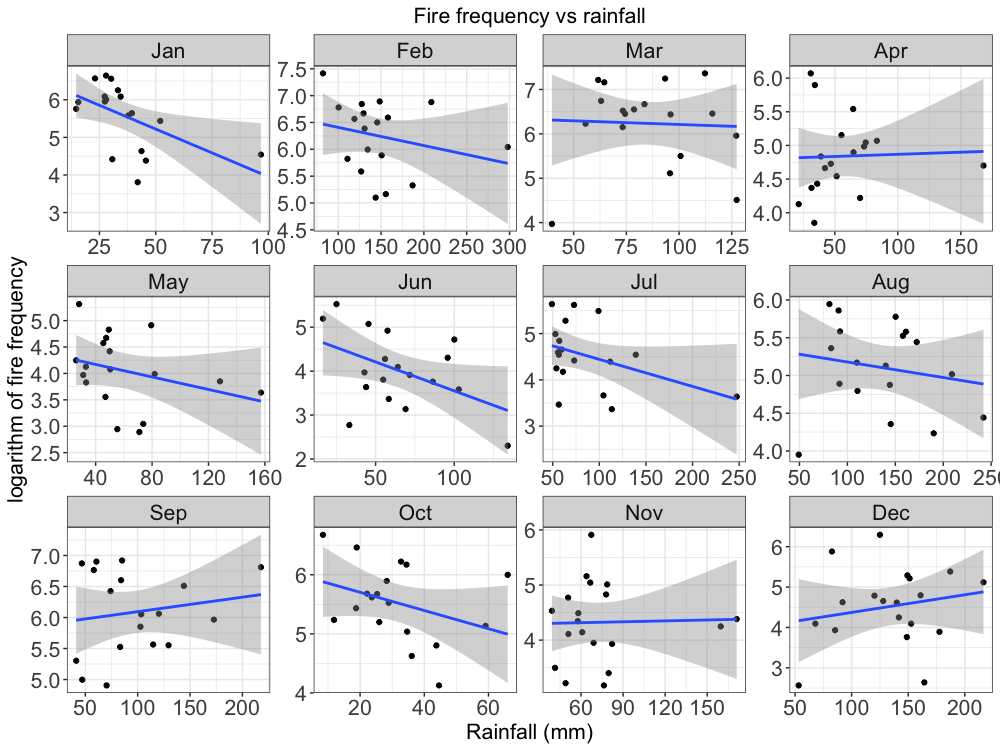

Supplement: S2 Fig — Scatter-plots of monthly relationships between mean rainfall and fire frequency. Graphs show different relationship for each month suggesting the seasonality and effect of unknown variables. (TIF) [file pone.0291800.s002.tif]
